# Rheology for Wood Plastic Composite Extrusion Part 2: Process Simulation and Experimental Verification

**DOI:** 10.3390/polym18060744

**Published:** 2026-03-19

**Authors:** Krzysztof J. Wilczyński, Kamila Buziak, Andrzej Nastaj, Adrian Lewandowski, Krzysztof Wilczyński

**Affiliations:** 1 Hanplast Ltd., 85-862 Bydgoszcz, Poland; wilczynski_k@wp.pl; 2 The Faculty of Mechanical and Industrial Engineering, Polymer Processing Institute, Warsaw University of Technology, 02-524 Warsaw, Poland; kamila.buziak@pw.edu.pl (K.B.); andrzej.nastaj@pw.edu.pl (A.N.); adrian.lewandowski@pw.edu.pl (A.L.)

**Keywords:** wood plastic composites, rheology, extrusion, simulations, viscosity

## Abstract

Rheological data of wood plastic composites (WPCs) are not readily present in many of the common scientific databases. For this reason, designing the processing of WPCs, e.g., extrusion, is difficult or even impossible, and it is often necessary to conduct research on your own to obtain the proper data. In the first part of the paper, studies of WPCs’ rheology have been performed in laboratory and production conditions. Tests in laboratory conditions have been conducted based on High-Pressure Capillary Rheometry (HPCR), using the Melt Flow Index (MFI). Tests in production conditions (on-line) have been performed by measuring the extrusion die pressure and extrusion throughput. The MFI’s viscosity and on-line viscosity results have been assessed against those of HPCR. In the second part of the paper, the viscosity data and models have been used for extrusion process simulations. Experimental studies of the process have been performed, and the experimental results have been used for evaluating the models applied. It was found that the two-point MFI method of determining viscosity and the on-line tests may be a reasonable alternative in the absence of HPCR data. The MFI method using the power-law model is fast and easy to apply and allows for analytical solutions to many processing problems. A significant advantage of on-line tests is that they are performed under real flow conditions of the tested material rather than laboratory conditions that do not take into account the material processing history.

## 1. Introduction

Common (pure) polymers are, in general, well known, and their material characteristics can be found in available databases, e.g., [1,2,3,4]. However, material data, e.g., rheological ones, of polyblends, polymer composites, or filled polymers are lacking in databases. They are also missing in the basic literature on the subject [5,6,7,8].

Rheological data are the basis for the good design of polymer processing, e.g., extrusion [9,10,11,12]. Rheological property tests are expensive and time consuming. Moreover, they are often carried out in conditions (temperature, pressure, shear rate) distant from those of processing. Therefore, cheap and fast methods for determining rheological data and methods implemented under processing conditions are sought [10,11,13,14].

The lack of rheological characteristics for advanced polymers is a serious problem in polymer processing and restricts the industrial use of available modeling (design) procedures (CAD/CAE software) [15,16,17,18]. This is shown, for example, by the authors’ studies on the extrusion process of polyblends [19,20,21,22,23,24], wood–plastic composites [25,26,27,28], or on modeling the injection molding [29,30].

In the first part of the paper [31], the studies investigating the rheology of WPCs have been performed in laboratory and production conditions. Tests in laboratory conditions have been conducted based on the High-Pressure Capillary Rheometry (HPCR), using the Melt Flow Index (MFI). Tests in production conditions (on-line) have been performed by measuring the extrusion die pressure and extrusion throughput. The MFI’s viscosity and on-line viscosity have been assessed against the background of the HPCR’s viscosity.

It was concluded that the MFI method of determining viscosity and the on-line tests may be a reasonable alternative to HPCR measurements. And, it was postulated to check this concept on the basis of theoretical and experimental studies of the extrusion process.

In this part of the paper, the viscosity data and models [31] have been used for extrusion process simulations. Experimental studies of the process have been performed, and the experiment results have been used for evaluating the models applied.

## 2. WPCs Extrusion

WPCs are resistant to moisture, can replace wood, and are therefore broadly applied in the economy. WPCs based on the HDPE, PP, and PVC are of fundamental importance [6,7,8,32,33,34,35,36]. Due to the widespread use of WPC profiles, the primary processing technique is extrusion.

Extrusion is the basic and most mass-produced technology for polymer processing. It can be performed in single screw or twin screw extruders with gravity or metered feed.

Single-screw extrusion with a typical gravity feed is relatively well understood. Tadmor et al., based on studies examining the melting of polymers, developed the first model of the process [37,38,39], which was followed by several others, e.g., NEXTRUCAD developed by Vlachopoulos [40], REX developed by Potente [41], and SSEM developed by Wilczyński [42]. Work on modeling single-screw extrusion was summarized, e.g., by Altinkaynak [43].

Twin-screw extrusion is much less well known than single-screw extrusion. Of fundamental importance here are the works of White et al., who, based on studies of the flow and melting of polymers, developed the first computer model of co-rotating extrusion—Akro-Co-Twin [44,45,46]. Subsequently, Potente developed the SIGMA program [47,48], and Vergnes developed the Ludovic program [49]. Work on the counter-rotating process was initiated by White et al., who, based on studies of the flow and melting of polymers, developed the first model of counter-rotating extrusion—Akro-Counter-Twin [50,51,52,53]. Research continued by Wilczyński et al. resulted in the TSEM program [20,54,55].

Single-screw extrusion with a metered feed has been very poorly understood until recently. The authors, based on experimental studies [56], proposed a melting model for this process [57] and then developed a global model of the whole process [58]. The model was extended to unconventional screw designs [59,60] and the extrusion of polyblends [19,20,21,22,23].

Knowledge on the processing and rheology of WPCs is very limited. The primary literature sources are still books by Mohanty [6], Klyosov [7], Oksman and Sain [8] and papers by Li and Wolcott [61,62,63], Xiao and Tzoganakis [64,65,66,67], and Vlachopoulos and Hristov [68,69]. Recently, good review papers have been written by Farul [70], Chan [71], Khan [72], Yadav [73], Ramesh [74], Elsheikh [75], Sun [76], and Mital’ová [77]. This was summarized by the authors [36].

Very recently, valuable studies on evaluating the melt viscosity in polymer extrusion were performed [78,79,80]. Pereira et al. [78] developed a gray-box soft sensing solution to predict the melt viscosity in real time, which combines physics-based knowledge with machine learning. They also proposed [79] a nonlinear model predictive control framework that enables the direct, real-time control of melt viscosity using non-invasive feedback from a deep neural network-based soft sensor. Houhat et al. [80] developed an experimental method to monitor the viscosity of polymer materials at high and varying temperatures by using ultrasound. This method allows us to measure the ultrasonic and rheological properties of a sample simultaneously and in real time.

Valuable research has also been carried out on the quality of wood composite products [81,82]. Pashazadeh et al. [81] elaborated a general procedure for combining material functions and numerical modeling to predict the orientation of highly filled wood polymer composites (WPCs) in a single-screw extrusion. They also proposed [82] a method for mapping surface defects in highly filled wood polymer composites (WPCs) in single-screw extrusion via inline optical spectral analysis.

Designing WPCs’ extrusion requires in-depth knowledge of the material flow mechanism in this process. Wilczyński [25] conducted studies on the extrusion of PP-based WPCs, which showed that the melting and flow in this case differ significantly from that of pure PP. It was found that solid conveying and material melting depended on the wood content and extruder operating conditions. The contiguous solid melting (CSM) mechanism, that is, the Tadmor mechanism [39], was not observed for materials with a higher filler content. However, it was observed for materials with a lower filler content—below 50%. Thus, it was concluded that for a lower filler content, classical two-dimensional melting occurs, while for a higher content, one-dimensional melting is observed. Based on this research, the first global model of WPCs extrusion has been developed [26]. And this model has been used in these studies.

## 3. Material

The WPC material PP copo inj 4, manufactured by Beologic company (Sint-Denijs, Belgium) and composed of polypropylene (PP) and 25% filler (WF), has been used in this paper. The material characteristics have been presented in the first part of the paper [31].

## 4. Rheological Models

WPCs are non-Newtonian, shear-thinning materials. Their viscosity lowers when the shear rate and temperature rise but grows with an increase in WF content. They may have yield stress and may slip when flowing. The slip velocity rises with shear rate, which promotes the formation of plug flow. A higher content of WF also promotes a plug flow. A comprehensive review on the rheology and processing of WPCs has been presented in [26,28,36].

In the first part of the paper [31], rheological studies of WPCs were conducted in laboratory settings and on-line. Classical High-Pressure Capillary Rheometry (HPCR) measurements using the capillary rheometer RG-25 and Melt Flow Index (MFI) measurements using the Melt Indexer MI-2 were performed [83,84]. Both instruments were manufactured by Goettfert company (Buchen, Germany). Tests in production conditions were conducted by measuring the extrusion throughput and die pressure.

The accuracy of MFI measurements is subject to errors that increase the viscosity value, which results from the use of a short capillary and the omission of the Bagley correction. It was observed that the MFI-based viscosity is approximately 20–30% higher than the HPCR reference. However, despite these discrepancies, it should be noted that the MFI measurements well reflect the dependence of viscosity on shear rate. The accuracy of on-line measurements is also subject to errors that raise the viscosity, which results from the omission of the Bagley correction. However, the MFI method using the power-law model is fast and easy to apply and allows for analytical solution of many processing problems. A significant advantage of on-line tests is that they are performed under real flow conditions of the tested material rather than under laboratory conditions that do not take into account the material processing history.

Finally, it was concluded that the two-point MFI method of determining viscosity and the on-line tests may be a reasonable alternative in the absence of HPCR data. And, it was postulated to check this concept on the basis of theoretical simulation and experimental testing of the extrusion process.

The results of rheological measurements have been described by rheological models. The results of HPCR measurements (corrected by Bagley and Rabinowitsch corrections) have been described using the Klein equation, which is typically applied in modeling the extrusion process [12,39,40]. The results of MFI and on-line measurements (corrected by Rabinowitsch correction) have been described by the Ostwald–de Waele model. It is easy to implement and consists of linear regression (in a double-logarithmic coordinate system) of the measurements data using the power-law equation.

**Figure 1:**
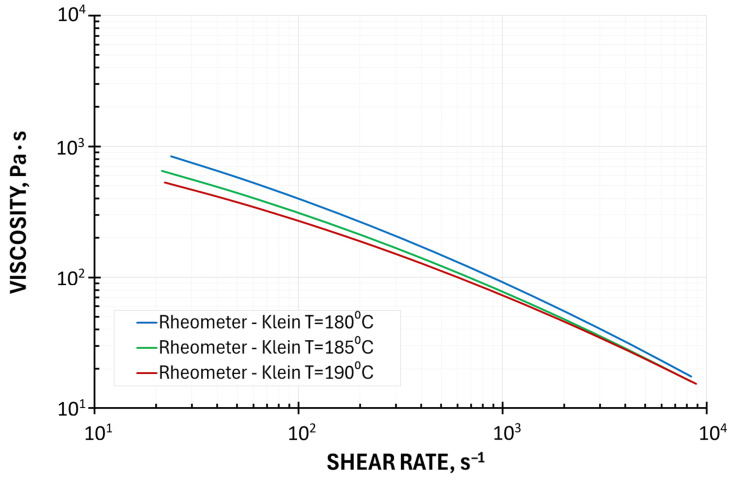
Viscosity curves by High-Pressure Capillary Rheometry (with Rabinowitsch and Bagley corrections) based on the Klein model.

**Figure 2:**
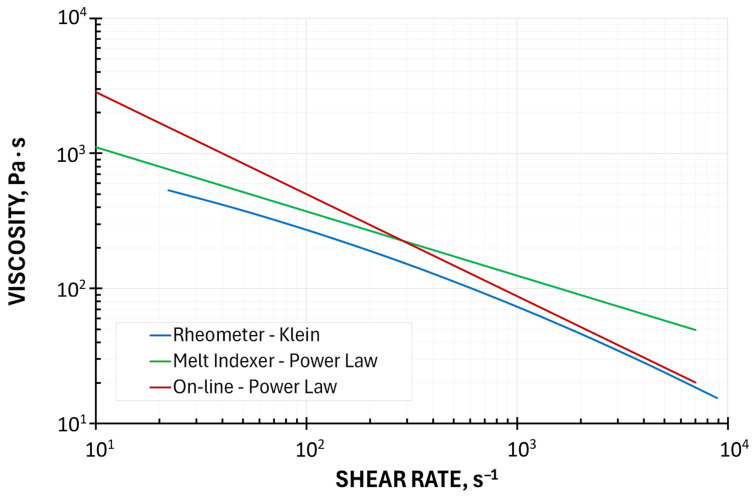
Viscosity curves by Melt Flow Index and on-line measurements (with Rabinowitsch corrections) based on the Ostwald–de Waele model compared to viscosity curve by High-Pressure Capillary Rheometry (at temperature T = 190 °C).

The graphical representations of the models used are presented in Figure 1 and Figure 2. It is seen from Figure 2 that the MFI viscosity curve is almost parallel shifted to the HPCR curve, toward higher values. The viscosity curve by on-line measurements at high shear rates is very close to the HPCR curve, while at low shear rates, the differences are very large. However, such low shear rates are beyond the processing range. So, it can be concluded that in the shear rate range 10^2^–10^3^ s^−1^, occurring in the extrusion process, the MFI viscosity and the viscosity by on-line measurements can be an alternative to HPCR measurements.

It is worth adding here that Le Moigne [85] and Polychronopoulos [86] showed that the viscosity of composites may be calculated by Einstein/Batchelor or Krieger/Dougherty equations, knowing the matrix viscosity and the filler content.

## 5. Experimental Section

**Figure 3:**
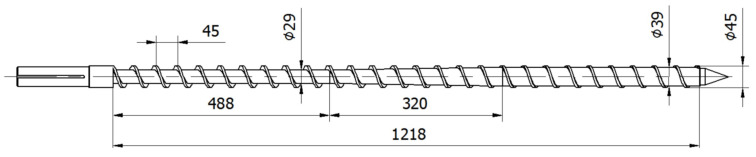
Screw geometry.

An experiment has been carried out with the use of a single screw extruder. A universal three-sectional screw of diameter D_SCR_ = 45 mm, length/diameter proportion L_SCR_/D_SCR_ = 27, and compression parameter (the ratio of the channel height in the first section to the channel height in the third section) CR = H_FEED_/H_METER_ = 8/3 has been used (Figure 3). The extrusion die was equipped with a flat mouthpiece of width W = 20 mm, height H = 2 mm, and length L = 80 mm, as presented in the first part of the paper [31]. Various screw speeds have been set: N_1_ = 30 rpm, N_2_ = 50 rpm, and N_3_ = 70 rpm. The barrel temperature has been set in subsequent zones as T_BARR_I_ = 180 °C, T_BARR_II_ = 180 °C, T_BARR_III_ = 190 °C, and T_BARR_IV_ = 190 °C, and the die temperature has been set as T_DIE_ = 190 °C.

**Figure 4:**
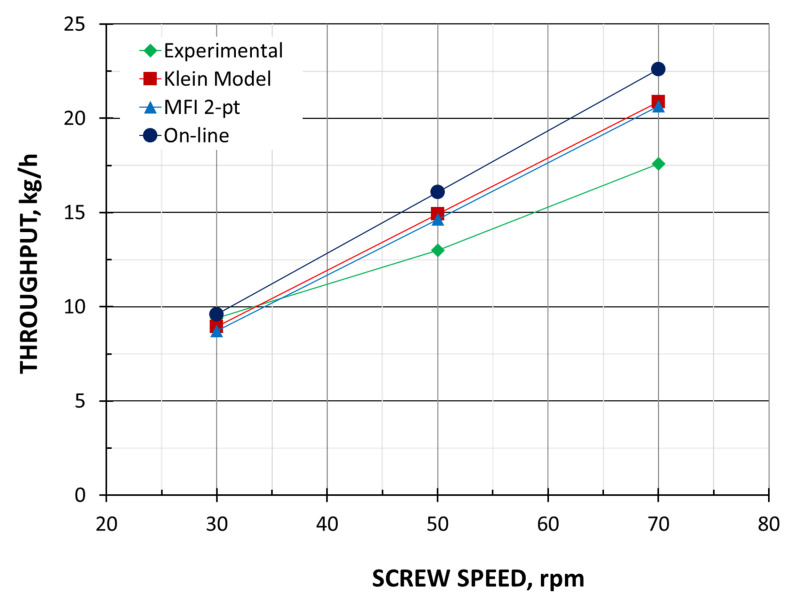
Extrusion throughput.

**Figure 5:**
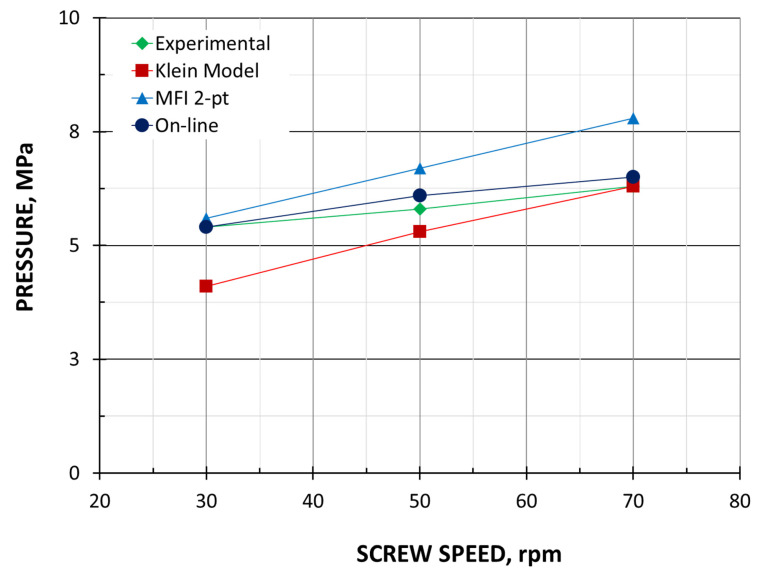
Die pressure.

**Figure 6:**
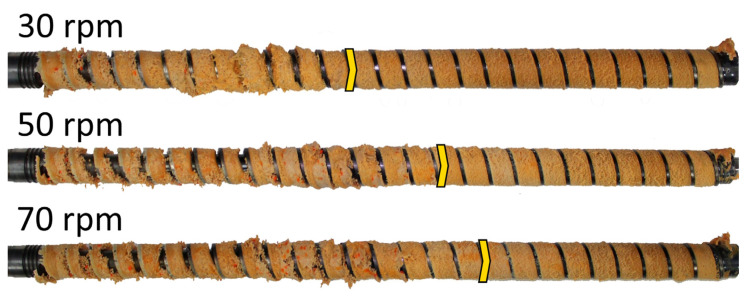
Screws removed from an extruder (the arrows indicate the end of melting).

The extrusion throughput (flow rate) was evaluated by measuring the mass of the material flowing out of the extruder die at a given time (gravimetrically, in steady state; 5 samples every 15 s), and the pressure was measured in the extruder die (P_DIE_) using a pressure sensor (manufactured by Dynisco, Franklin, MA, USA) placed 0.8 m before the end of the die (in the same time interval as the flow rate). A “screw pulling-out technique” has been used to evaluate the polymer melting. This well-known technique has been presented elsewhere, e.g., [25]. The results of the experiment are presented in Figure 4, Figure 5 and Figure 6.

It is seen that the extrusion throughput (Figure 4) and die pressure (Figure 5) increase almost linearly as the screw speed increases. The melting length (Figure 6) also increases with as the screw speed increases. This is because the material spends less time in the barrel (the residence time is shorter). In each case, however, the material is completely melted in the extruder and flows into the die in a molten state.

## 6. Simulations

### 6.1. Simulation Software

The MULTI-SCREW system developed by the authors has been used in the study [12]. It consists of four subsystems: the GSEM program (Global-Screw Extrusion Model) for modeling the single-screw extrusion, TSEM program (Twin-Screw Extrusion Model) for modeling the twin-screw extrusion, GASEO program (Genetic Algorithms Screw Extrusion Optimization) for extrusion process optimization, and the recently developed GASES program (Genetic Algorithms Screw Extrusion Scale-Up) for scaling up the extrusion process.

The GSEM software applied here is a program designed to simulate the single-screw extrusion process with both flood feeding and metered feeding. The program allows for the simulation of extrusion with conventional and non-conventional screws equipped with mixing and shearing elements. The extrusion dies may have channels of various cross-sections. The program simulates an operation of the extrusion system to predict the extrusion throughput (at flood feeding), the screw filling (in the case of metered feeding), the pressure and temperature distributions along the screw/die length, the melting of the polymer, the power consumption, and many other process parameters. The simulation input data are the material characteristics, e.g., thermal and rheological characteristics, the screw and die geometry, and the process operating data (screw speed, barrel and die temperature). Based on the further studies [26,28,36], the system has been developed to simulate the WPCs’ extrusion.

**Figure 7:**
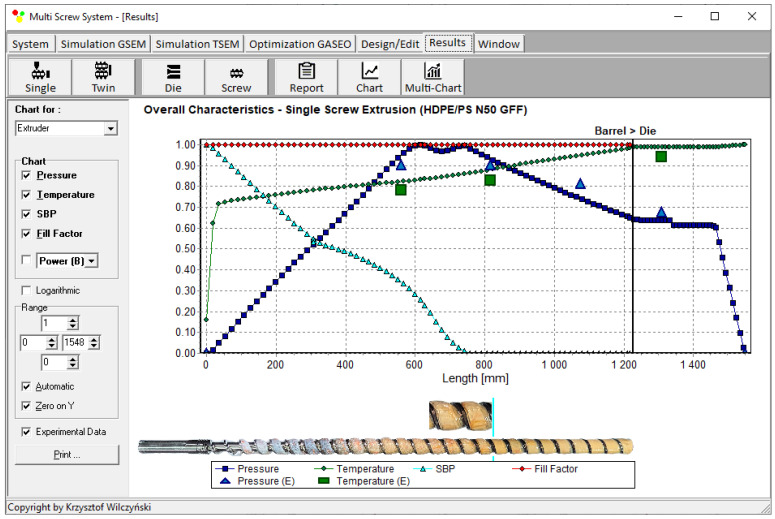
Overall characteristics of flood fed single-screw extrusion, computations by the (GSEM) program and experimental data: SBP—solid bed profile, E—experiment [12].

An example of a simulation is presented in Figure 7. The overall process characteristics (dimensionless) include the pressure and temperature distributions, the screw-filling distribution, and the polymer melting progress represented by the proportion of the polymer solid bed width X to the channel width W, i.e., the solid bed parameter SBP = X/W. The simulations are complemented by the results of experimentation, which include the pressure and temperature measurements, and the picture of the screw pulled out of the barrel, presenting the melting progress of the material.

### 6.2. Simulation Results

The assessment of the usefulness of the concepts discussed in this paper for describing the rheological properties of polymers in designing the process was conducted based on simulations of the three key extrusion process characteristics—that is, the process throughput, the pressure in the die, and the polymer melting process. The first requirement for a good extrusion process design is ensuring adequate pressure within the extruder, which is sufficient to allow the polymer to be extruded through the die. The second requirement for a good extrusion process design is ensuring that the polymer is fully molten in the extruder and does not flow unmolten into the die. Of course, an important parameter is the extrusion throughput.

The simulations were performed based on the material, geometry and operating data such as those used in the experimental studies. Three types of rheological data were used: viscosity data based on the HPCR testing, viscosity data based on the MFI testing, and viscosity data based on the on-line measurements (Figure 2).

**Figure 8:**
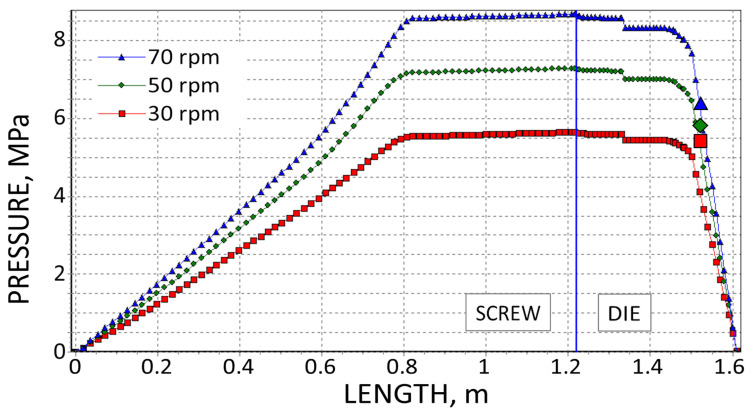
Pressure simulations using viscosity data by High-Pressure Capillary Rheometry and experimental die pressure data (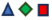).

**Figure 9:**
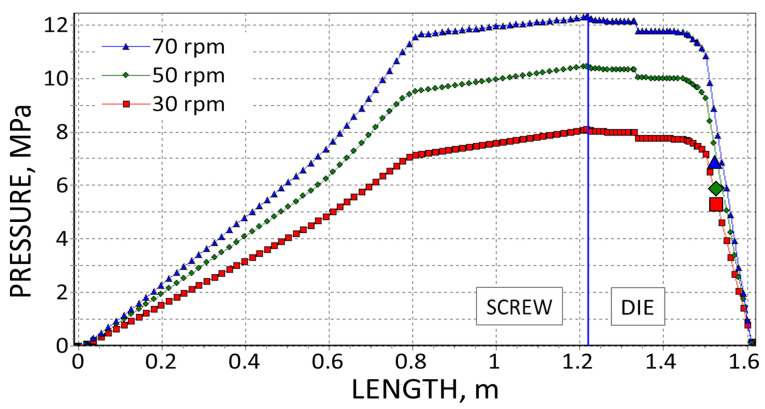
Pressure simulations using viscosity data by Melt Flow Index and experimental die pressure data (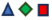).

**Figure 10:**
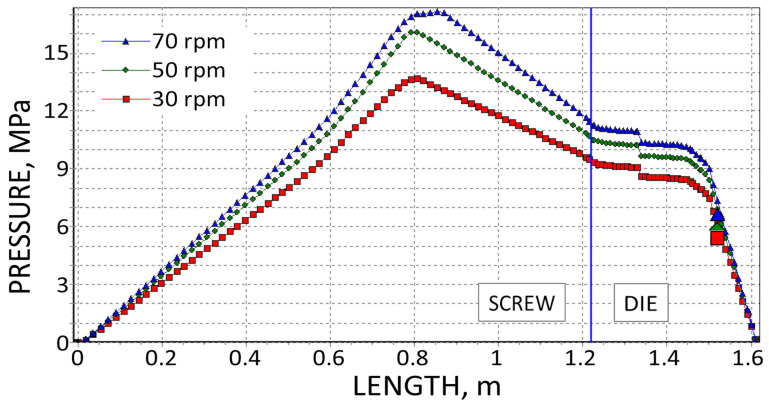
Pressure simulations using viscosity data by on-line measurements and experimental die pressure data (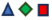).

**Figure 11:**
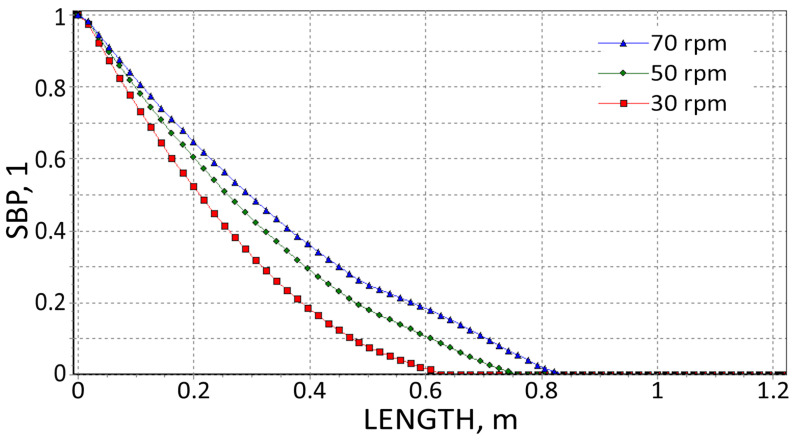
SBP profiles using viscosity data by High-Pressure Capillary Rheometry.

**Figure 12:**
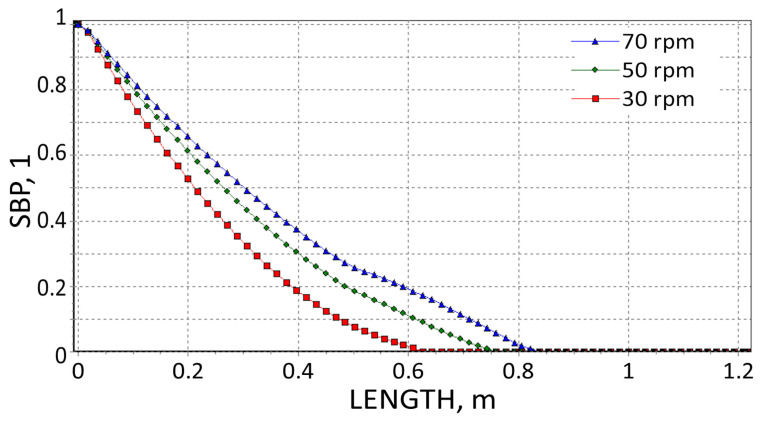
SBP profiles using viscosity data by Melt Flow Index.

**Figure 13:**
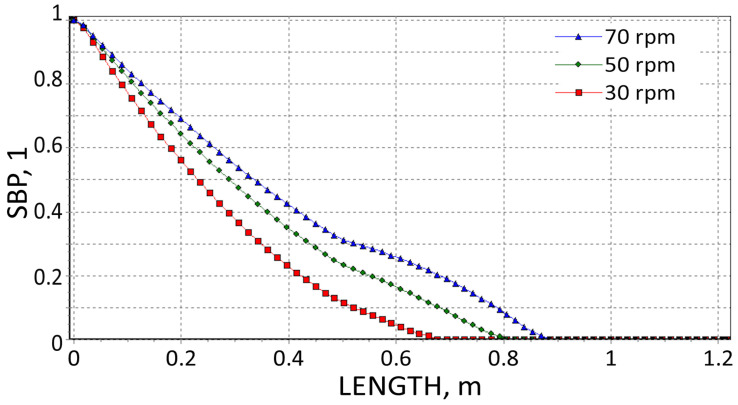
SBP profiles using viscosity data by on-line measurements.

The results are presented in Figure 8, Figure 9, Figure 10, Figure 11, Figure 12 and Figure 13. The pressure simulations are presented in Figure 8, Figure 9 and Figure 10, and simulations of polymer melting, i.e., SBP distributions (solid bed parameter SBP = X/W), are depicted in Figure 11, Figure 12 and Figure 13.

The extrusion throughput and die pressure simulations were compared with the experimental data in Figure 4 and Figure 5. The simulations of polymer melting can be compared via the experiment shown in Figure 6.

In general, the computations of extrusion throughput are consistent with the experimental results. As in the experiment, the extrusion throughput increases almost linearly with increasing screw speed for each method used. At low screw speeds, the calculations are consistent with the experiment. When the screw speed rises, the discrepancies between the calculations and the experiment increase, but these are within 10–20%. The results obtained with the use of MFI viscosity data and the results obtained with the use of HPCR viscosity data are consistent. The results based on the on-line measurements differ slightly.

The calculations of die pressure are, in general, also consistent with the experimental results. As in the experiment, the die pressure increases almost linearly with the increasing screw speed for each method used. The discrepancies between the calculations and the experiment are within 10–20%. The results based on the on-line measurements are fully consistent with those from the experiment. The results obtained with the use of MFI viscosity data are parallelly shifted toward higher values compared to the HPCR data, which results from not taking into account the Bagley correction.

The relative simulation error for the extrusion throughput and die pressure may be evaluated as

(1) $$\delta_{G_{i}}=\frac{G_{sim,i}-G_{exp,i}}{G_{exp,i}}\times 100\%$$

(2) $$\delta_{P_{i}}=\frac{P_{sim,i}-P_{exp,i}}{P_{exp,i}}\times 100\%$$

where δ is the relative error, G is the throughput, P is the die pressure, and *i* ∈ {30, 50, 70 rpm}.

These are presented in Table 1 and Table 2.

The simulations of pressure distribution in the extruder, which are shown in Figure 8, Figure 9 and Figure 10, obtained on the basis of three different calculation methods, differ significantly, which is mainly due to the differences in extrusion throughput and viscosity data. Although in each case, when the screw speed rises, the pressure increases.

It is interesting that the melting simulations are consistent with the results from the experiment, and the results obtained using different methods are convergent. The melting length (Figure 11, Figure 12 and Figure 13) rises as the screw speed increases in each case. This is because the material spends less time in the machine (the residence time is shorter). Also, in each case, the material is completely melted in the extruder and flows into the die in a molten state.

## 7. Conclusions and Future Trends

Common polymers are, in general, well known, and their material characteristics can be found in the available databases. However, material data, e.g., rheological ones, of polyblends, polymer composites, or filled polymers are lacking in the same databases.

Therefore, an attempt was made to develop a technique to determine the rheological characteristics of polymeric materials based on simple and easily accessible measurements, in cases where such data are unavailable in the material databases, literature or manufacturer’s materials.

In the first part of the paper [31], the studies examining the rheology of WPCs have been performed in laboratory and production conditions. Tests in laboratory conditions have been conducted based on the High-Pressure Capillary Rheometry (HPCR), using the Melt Flow Index (MFI). Tests in production conditions (on-line) have been performed by measuring the extrusion die pressure and extrusion throughput. The MFI’s viscosity and on-line viscosity have been assessed against the background of the HPCR’s viscosity. It was concluded that the two-point method of determining viscosity based on the MFI measurements and the on-line tests may be a reasonable alternative to HPCR measurements. And, it was postulated to check this concept on the basis of theoretical and experimental studies of the extrusion process.

In this paper, the results of rheological studies (viscosity curves and models) [31] have been applied for extrusion process modeling (extrusion simulations). Experiments on extrusion have been performed, and their results have been used for evaluating the models applied.

It was concluded that the concepts of using the limited MFI measurements and on-line measurements can be applied in engineering for quickly evaluating the rheological properties of WPCs when available databases do not provide such data. Although these data are subject to errors, they always provide more information about the material being processed than the Melt Flow Index (MFI), which is not always provided by the producer.

It is worth noting that many issues of polymer processing can be solved by simple and fast calculations, the accuracy of which is often only slightly lower than computations by advanced CAD/CAE systems. Simulations are usually time consuming, expensive, and difficult, and they sometimes lead to erroneous results due to the lack of good input data (material, operation, and geometry) for calculations. There is a well-known saying “garbage in, garbage out”. Moreover, the effective use of CAD/CAE systems requires extensive knowledge of the issue under study and the system used as well as a solid understanding of rheology and thermodynamics. It is also important to remember that the computational system selected should always be appropriate to the complexity of the issue under study. In the absence of good input data, e.g., material data, the use of advanced CAD/CAE systems is unjustified. The future aim of our work is to develop an engineering system that would allow for the fast calculations of basic processing issues with engineering accuracy. This approach can be found in [11,14,87].

Summarizing, the rheological studies of WPCs and modeling of WPCs’ processing require solving some issues:

- Slip and yield stress should be considered when modeling the polymer processing. Slip effects can be removed from rheological measurements by Mooney correction. Slip effects in process modeling result in the flow rate increasing and the pressure decreasing. These have been discussed by the authors [88,89] and, for example, in [90,91,92,93,94].
- Slip effects in WPCs extrusion were discussed by the authors [28], and it was concluded that when slip appears, the flow rate (extrusion throughput) increases, while the pressure (die pressure) decreases, and melting is slower in this case, which may be caused by an increase in the flow rate. The GSEM model used here allows to take into account the slip effects; however, in this study, the slip effects were not included in the rheological model.
- The rheological data of WPCs are generally not available in the databases, especially when taking into account slip and yield stress. There are no reliable thermal data for WPCs, such as the heat of fusion, melting or softening point, etc. It is difficult to determine the melting point, and therefore, the concept of no-flow temperature is proposed to determine the onset of flow. Moreover, the effect of pressure on rheological properties is important. These issues were discussed in the literature, e.g., [95,96,97,98].
- Due to the lack of rheological data for WPCs, in-house research methods for the determination of viscosity curves by MFI tests and on-line tests are proposed. The two-point MFI method is fast and easy to apply and allows for the analytical solution of many problems related to polymer processing. Production tests have a significant advantage because they are conducted under real flow conditions of the tested material rather than under laboratory conditions that do not take into account the material processing history.
- An error was observed in the MFI measurements due to the use of a short capillary and the omission of the Bagley correction. It seems reasonable to look for a computation method that accounts for these systematic deviations.
- An error was also observed in the on-line measurements, resulting from a failure to include the Bagley correction in the calculations. It seems reasonable also in this case to look for a computation method that accounts for these systematic deviations.
- Errors observed in the on-line measurements due to the omission of the Bagley correction in the calculations may be removed by utilizing a dual-channel measurement die (of various channel lengths) and pressure measurement in each channel. This design eliminates the influence of inlet losses in the measurements and allows for the introduction of the Bagley correction into the calculations.
- The concepts presented here may be applied to other polymeric materials, for example, polymer composites, polyblends or filled polymers.

## Figures and Tables

**Table 1:** Simulations and experimental data for the extrusion throughput.

Screw Speed	Throughput						
	Experimental	Klein Model	Error	MFI 2-pt (1.2–15 kg)	Error	On-Line	Error
N, rpm	*G_exp_*, kg/h	*G_Klein_*, kg/h	*δ_G_Klein_*, %	*G*_2_*_pt_*, kg/h	*δ_G__*_2_*_pt_*, %	*G_On-line_*, kg/h	*δ_G_On-line_*, %
30	9.4	9.0	4.8	8.7	7.1	9.6	2.1
50	13.0	14.9	14.8	14.6	12.6	16.1	23.8
70	17.6	20.9	18.7	20.6	17.3	22.6	28.5

**Table 2:** Simulations and experimental data for the die pressure.

Screw Speed	Die Pressure						
	Experimental	Klein Model	Error	MFI 2-pt (1.2–15 kg)	Error	On-Line	Error
N, rpm	*P_exp_*, MPa	*P_Klein_*, MPa	*δ_P_Klein_*, %	*P*_2_*_pt_*, MPa	*δ_P__*_2_*_pt_*, %	*P_On-line_*, MPa	*δ_P_On-line_*, %
30	5.4	4.1	24.1	5.6	3.7	5.4	0.0
50	5.8	5.3	8.6	6.7	15.5	6.1	5.2
70	6.3	6.3	0.0	7.8	23.8	6.5	3.2

## Data Availability

The original contributions presented in this study are included in the article. Further inquiries can be directed to the corresponding author.
